# Rickettsiae in Ticks, Japan, 2007–2011

**DOI:** 10.3201/eid1902.120856

**Published:** 2013-02

**Authors:** Gaowa, Norio Ohashi, Minami Aochi, Wuritu, Dongxing Wu, Yuko Yoshikawa, Fumihiko Kawamori, Toshiro Honda, Hiromi Fujita, Nobuhiro Takada, Yosaburo Oikawa, Hiroki Kawabata, Shuji Ando, Toshio Kishimoto

**Affiliations:** Author affiliations: University of Shizuoka Global Center of Excellence Program, Shizuoka, Japan (Gaowa, N. Ohashi, M. Aochi, Wuritu, D. Wu, Y. Yoshikawa, F. Kawamori);; Shizuoka Institute of Environment and Hygiene, Shizuoka (F. Kawamori);; Kagoshima Prefectural Institute for Environment Research and Public Health, Kagoshima, Japan (T. Honda);; Ohara General Hospital, Fukushima, Japan (H. Fujita);; Fukui University, Fukui, Japan (N. Takada); Kanazawa Medical University, Ishikawa, Japan (Y. Oikawa);; National Institute of Infectious Diseases, Tokyo, Japan (H. Kawabata, S. Ando);; Okayama Prefectural Institute for Environmental Science and Public Health, Okayama, Japan (T. Kishimoto)

**Keywords:** Rickettsiales, Rickettsia, rickettsiae, Anaplasma phagocytophilum, Ehrlichia, tickborne infections, spotted fever group, Japanese spotted fever, gltA, 16S rDNA, ompA, p44/msp2, p28/omp-1, bacteria, surveillance, epidemiology, Japan

**To the Editor:** Japanese spotted fever (JSF), caused by *Rickettsia japonica*, is the most prevalent tickborne infectious disease in Japan (*1*), occurring most frequently in central and western regions (http://idsc.nih.go.jp/idwr/CDROM/Main.html [in Japanese]). Cases of unknown fever with rickettsiosis-like symptoms not associated with JSF have been reported in JSF-endemic regions of Japan (*2*). Several spotted fever group (SFG) rickettsiae (*R. japonica*, *R. heilongjiangensis*, *R. helvetica*, *R. tamurae*, *R. asiatica*, *Candidatus* R. tarasevichiae) and other related *Rickettsia* spp. have been identified in Japan (*1*,*3*–*6*). Human infections with *R. heilongjiangensis* and *R. tamurae* have been confirmed (*3*,*5*), and *Anaplasma phagocytophilum* and *Ehrlichia chaffeensis*, known human pathogens, have been detected in ticks and deer in Japan. We conducted this study to determine the risk in central and western Japan for human exposure to ticks harboring SFG rickettsiae, *A. phagocytophilum*, or *Ehrlichia* spp. 

In 2007–2011, we collected 827 *Haemaphysalis*, *Amblyomma*, and *Ixodes* spp. ticks (392 adults, 435 nymphs) by flagging vegetation in the prefectures of Shizuoka, Mie, Wakayama, Kagoshima, Nagasaki (Goto Island), and Okinawa (the main island and Yonaguni Island) (Technical Appendix Figure 1). We extracted DNA from the salivary glands of each tick and performed PCR to amplify *gltA*, 16S rDNA, and *ompA* of SFG rickettsiae. To detect *A. phagocytophilum* and *Ehrlichia* spp., we performed nested PCR targeting the *p44/msp2* and *p28/omp-1* multigenes, respectively.

PCR *gltA* screening revealed SFG rickettsiae in 181 (21.9%) of the 827 ticks (Table). We obtained nearly full-length (1.1-kb) *gltA* sequences and classified them into 5 groups by phylogenetic analyses (Technical Appendix Figure 2). Sequences for groups 1 (prevalence 1.0%) and 2 (prevalence 3.2%) were identified as *R. japonica* YH (GenBank accession no. AP011533) and *R. tamurae* (GenBank accession no. AF394896), respectively (Table). Group 3 (prevalence 15.1%) sequences were identical to that of *Rickettsia* sp. LON (GenBank accession no. AB516964). The sequence for group 4 (prevalence 1.6%) was closely related to that for *R. raoultii* strain Khabarovsk (98.8% similarity), and a part of the sequence (342 bp) was identical to that of *Rickettsia* sp. Hf 151 (GenBank accession no. AB114815). Group 5 consisted of 4 newly identified rickettsiae (Technical Appendix Figure 2). Of these 4 rickettsiae, 3 (Mie311, Goto13, and Mie334) were closely related to *R. raoultii* strain Khabarovsk (98.0% identity) and 1 (Mie201) was similar to *Candidatus* R. principis (99.7% identity). 

**Table:** PCR survey results for *Haemaphysalis*, *Amblyomma*, and *Ixodes* spp. ticks tested for rickettsiae, central and western Japan, 2007–2011*

Tick species	No. ticks tested	Total no. (%) ticks positive	No. (%) ticks positive for						
			*Rickettsia gltA*, by species group†					*A. phagocytophilum p44/msp2*‡	*Ehrlichia p28/omp-1*§
			Group 1	Group 2	Group 3	Group 4	Group 5		
*H. formosensis*	224	6 (2.7)	1 (0.4)	0	0	0	5 (2.2)	18 (8)	0
*H. hystricis*	97	19 (19.6)	6 (6.1)	0	0	13 (13.4)	0	0	0
*H. longicornis*	294	119 (40.5)	0	0	119 (40.5)	0	0	2 (0.7)	1 (0.4)
*H. flava*	55	6 (10.9)	0	0	2 (3.6)	0	4 (7.3)	0	0
*H. kitaokai*	10	0	0	0	0	0	0	0	0
*H. megaspinosa*	18	4 (22.2)	0	0	4 (22.2)	0	0	1 (5.6)	0
*H. cornigera*	11	1 (9.1)	1 (9.1)	0	0	0	0	0	0
*A. testudinarium*	112	26 (23.2)	0	26 (23.2)	0	0	0	3 (2.7)	1 (0.9)
*A. geoemydae*	1	0	0	0	0	0	0	0	0
*I. ovatus*	5	0	0	0	0	0	0	1 (20.0)	0
Total	827	181 (21.9)	8 (1.0)	26 (3.1)	125 (15.1)	13 (1.6)	9 (1.1)	25 (3.0)	2 (0.2)

*DNA was extracted from the salivary glands of each tick by using the DNeasy Mini Kit (QIAGEN Sciences, Germantown, MD, USA) and used as a template for PCR. The newly identified sequences of *gltA,* 16S rDNA, *ompA*, *p44/msp2*, and *p28/omp*-1 in this study were deposited into GenBank under accession nos. JQ697880–JQ697959. *A. phagocytophilum, Anaplasma phagocytophilum.* †The PCR primers used, *gltA*–Fc (5′-CGAACTTACCGCTATTAGAATG-3′) and *gltA*–Rc (5′-CTTTAAGAGCGATAGCTTCAAG-3′), were designed in this study. Groups: 1, *Rickettsia japonica* YH (GenBank accession no. AP011533); 2, *R. tamurae* (GenBank accession no. AF394896); 3, *Rickettsia* sp. LON-13 (GenBank accession no. AB516964); 4, *Rickettsia* sp. Hf151; 5, other rickettsiae. ‡PCR primers of p3726 (5′-GCTAAGGAGTTAGCTTATGA-3′), p3761 (5′-CTGCTCT[T/G]GCCAA(AG)ACCTC-3′, p4183 (5′-CAATAGT[C/T]TTAGCTAGTAACC-3′), and p4257 (5′-AGAAGATCATAACAAGCATTG-3′) were used for detection of *p44/msp2*. §PCR primers conP28-F1 (5′-AT[C/T]AGTG[G/C]AAA[A/G]TA[T/C][A/G]T[G/A]CCAA-3′), conP28-F2 (5′-CAATGG[A/G][T/A]GG[T/C]CC[A/C]AGA[A/G]TAG-3′), conP28-R1 (5′-TTA[G/A]AA[A/G]G[C/T]AAA[C/T]CT[T/G]CCTCC-3′), and conP28-R2 (5′-TTCC[T/C]TG[A/G]TA[A/G]G[A/C]AA[T/G]TTTAGG-3′) were used to detect *p28/omp-1*.

We further analyzed the 16S rDNA and *ompA* in *gltA*-positive tick samples. The 16S rDNA and *ompA* for group 1 samples shared 100% identity with 16S rDNA and *ompA* of *R. japonica* YH (AP011533). The 16S rDNA of group 2 was identical to that of *R. tamurae* (AY049981). In groups 3–5, some of the specific amplicons in 16S rDNA or *ompA* could be detected; their sequences were confirmed to be similar (but not identical) to those of several known rickettsial sequences.

We amplified the *p44/msp2* amplicons of *A. phagocytophilum* from 25 (3%) of 827 ticks (Table). By cloning (TA Cloning Kit; Life Technologies, Carlsbad, CA, USA) and sequencing these amplicons, we obtained and identified 60 new TA-clone sequences (366–507 bp) for *p44/msp2* (GenBank accession nos. JQ697880–JQ697950); these sequences may include a potentially novel *Anaplasma* species. (*7*). *Ehrlichia p28*/*omp-1* was detected from 2 (0.2%) of the 827 ticks. Of 5 TA-clone sequences (284–315 bp) obtained from the 2 ticks, 2 from an *A. testudinarium* tick (GenBank accession nos. JQ697886 and JQ697887) shared 83.3%–86.7% similarity with *E. ruminantium* Gardel Map-1 (GenBank accession no. YP196842), and 3 from an *H. longicornis* tick (GenBank accession nos. JQ697888–JQ697890) showed the closest relationship to *E. ewingii omp-1–15* (67%–73% similarity; GenBank accession no. EF116932).

We identified the tick species associated with *R. japonica* as *H. formosensis*, *H. hystricis*, and *H. cornigera*, and another study reported an association with *Dermacentor taiwanensis*, *H. flava*, *H. longicornis*, and *I. ovatus* (*4*). In our study and previous studies, the tick species associated with *A. phagocytophilum* in Japan were identified as *H. formosensis*, *H. longicornis*, *H. megaspinosa*, *A. testudinarium*, *I. ovatus*, and *I. persulcatus* (*8*). Thus, it appears that 3 tick species (*H. formosensis*, *H. longicornis*, and *I. ovatus*) are associated with *R. japonica* and *A. phagocytophilum*. 

In addition, in an *H. formosensis* tick, we detected an SFG rickettsia that is closely related to *R. raoultii*, the etiologic agent of *Dermacentor*-borne necrosis erythema and lymphadenopathy in Europe and Russia (*9*). We detected *Candidatus* R. principis in *H. flava* in Japan; this species was previously detected in *H. japonica douglasi* and *H. danieli* ticks in Russia and China, respectively, (*10*). And, we found a high prevalence of *R. tamurae* in *A. testidinarium* ticks; Imaoka et al. (*5*) recently reported that *R. tamurae* causes local skin inflammation without general JSP-like symptoms. We did not detect the human pathogen *E. chaffeensis*, but we identified 2 potentially new *Ehrlichia* species.

Our findings contribute to the known risks for exposure to *Rickettsia*-related pathogens in central and western Japan. Further studies may be required for the surveillance of additional pathogens, such as *Candidatus* Neoehrlichia mikurensis (*2*), which was recently recognized as a human pathogen.

Technical Appendix Phylogenetic classification of Rickettsia spp. gltA sequences detected in ticks during 2007–2011 in central and western Japan and locations of tick collection sites.
