# Supplementary material for: Rickettsiae in Ticks, Japan, 2007–2011
Source: Emerg Infect Dis. 2013 Feb;19(2):338–40. doi: 10.3201/eid1902.120856 (PMC3559048; doi:10.3201/eid1902.120856)
Supplement: Technical Appendix — Phylogenetic classification of Rickettsia spp. gltA sequences detected in ticks during 2007–2011 in central and western Japan and locations of tick collection sites. [file 12-0856-Techapp-s1.pdf]

Article DOI: http://dx.doi.org/10.3201/eid1902.120856

Rickettsiae in Ticks, Japan, 2007–2011

Technical Appendix

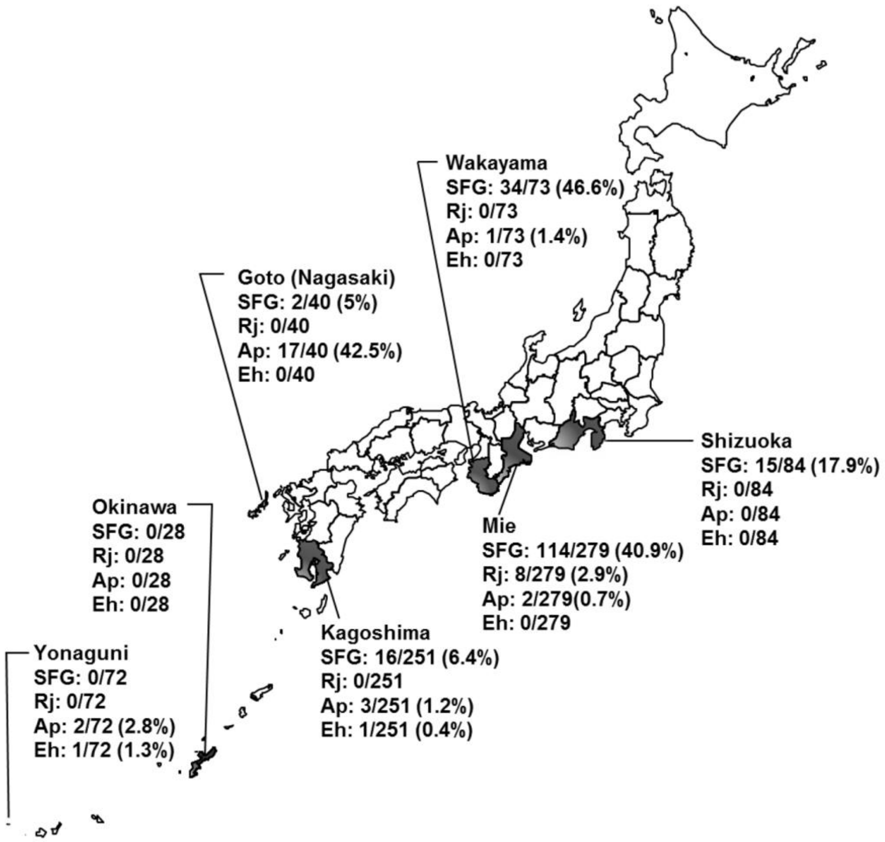

Figure 1. Prefectures where ticks were collected for a study of *Rickettsia* spp.–related pathogens in ticks in central and western Japan, 2007–2011. SFG, spotted fever group rickettsiae; Rj, *R. japonica*; Ap, *A. phagocytophilum*; Eh, *Ehrlichia* spp. Numbers indicate the number of positive ticks/number of ticks tested. The prevalence of respective *Rickettsiales* bacteria in each area is shown in parentheses.

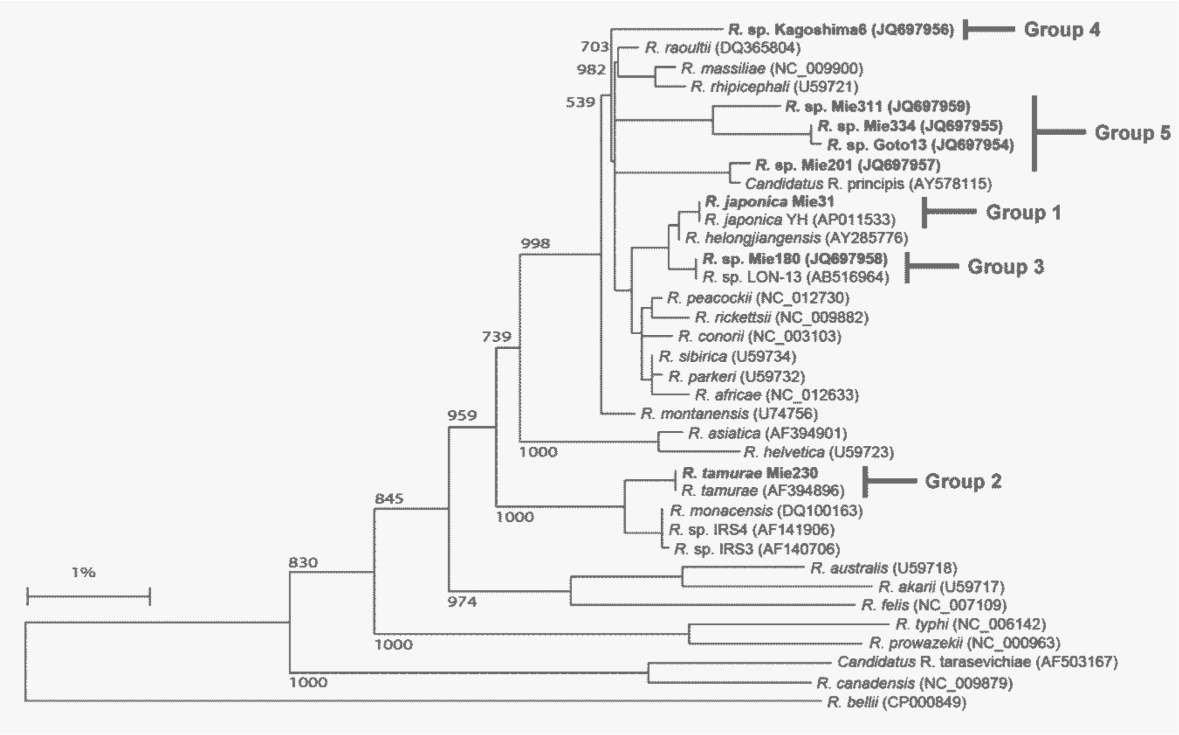

Figure 2. Phylogenetic classification of *Rickettsia spp. gltA* sequences detected in ticks inhabiting central and western Japan, 2007–2011. The tree, based on the *gltA* sequences (1,115–1,123 bp), was constructed by using the neighbor-joining method with 1,000 bootstrap resamplings. **Boldface** font indicates *Rickettsia* spp. identified in this study. Numbers in parentheses indicate GenBank accession numbers for the respective sequences. The scale bar indicates nucleotide substitutions per site.
